# Spatial transcriptome data from coronal mouse brain sections after striatal injection of heme and heme-hemopexin

**DOI:** 10.1016/j.dib.2022.107866

**Published:** 2022-01-22

**Authors:** Kevin Akeret, Michael Hugelshofer, Dominik J. Schaer, Raphael M. Buzzi

**Affiliations:** a Department of Neurosurgery, Clinical Neuroscience Center, Universitätsspital und University of Zurich, Frauenklinikstrasse 10, Zurich CH-8091 Switzerland; b Division of Internal Medicine, Universitätsspital and University of Zurich, Rämistrasse 100, Zurich CH-8091 Switzerland

**Keywords:** spRNAseq, spatial RNA sequencing, Hpx, hemopexin, Spatial RNA sequencing, Secondary brain injury, Intracerebral hemorrhage, Heme toxicity, Visium

## Abstract

Hemorrhagic stroke is a major cause of morbidity and mortality worldwide. Secondary mechanisms of brain injury adversely affect functional outcome in patients after intracranial hemorrhage. Potential drivers of intracranial hemorrhage-related secondary brain injury are hemoglobin and its downstream degradation products released from lysed red blood cells, such as free heme. We established a mouse model with stereotactic striatal injection of heme-albumin to gain insights into the toxicity mechanisms of free heme in the brain and assess the therapeutic potential of heme binding and biochemical neutralization by hemopexin. We defined the dose-dependent transcriptional effect of heme or heme-hemopexin exposure 24 h after injection by spatial transcriptome analysis of lesion-centered coronal cryosections. The spatial transcriptome was interpreted in a multimodal approach along with histology, magnetic resonance imaging, and behavioral data and reported in the associated research article “Spatial transcriptome analysis defines heme as a hemopexin-targetable inflammatoxin in the brain” [1].

The spatially resolved transcriptome dataset made available here is intended for continued analysis of free heme toxicity in the brain, which is of potential pathophysiological and therapeutic significance in the context of a wide range of neurovascular and neurodegenerative diseases.

## Specifications Table

**Table:** 

Subject	Neuroscience: General
Specific subject area	Secondary brain injury after intracranial hemorrhage, mouse model, spatial transcriptome analysis
Type of data	Table Image Figure
How the data were acquired	A mouse model with striatal injection of heme, hemopexin or vehicle control (saline) was used. Histological images were acquired on an AxioObserver (Zeiss) at a 10x magnification. mRNA extraction and library construction from cryosections using a Visium Spatial Slide (10X Genomics, Pleasanton, CA, USA) according to the manufacturer's instructions (User Guide CG000239, Rev A). Sequencing was performed on an Illumina NovaSeq 6000 (Illumina, San Diego, CA, USA) using a 28 bp + 120 bp paired-end sequencing mode. The mapping and counting of the reads to the reference genome GRCm38.p6 (mm10), as well as the alignment of the barcodes in each read with the 55-µm features in the spatial dimension was performed using Space Ranger 1.1.0. (10X Genomics, Pleasanton, CA, USA). Visualization, data processing and downstream analysis was performed using the Scanpy python package (version 1.4.4).
Data format	Raw Analyzed Filtered
Description of data collection	The spatial transcriptomes of coronal brain sections from mice 24h after striatal injection of 10 µL heme, heme-hemopexin or vehicle control (saline) solution were sequenced using the Visium workflow from 10X Genomics (Pleasanton, CA, USA).
Data source location	• Institution: Division of Internal Medicine, Universitätsspital and University of Zurich • City/Town/Region: Zurich • Country: Switzerland
Data accessibility	Repository name: Gene expression omnibus Permanent identification number: GSE182127 Direct link to the dataset: https://www.ncbi.nlm.nih.gov/geo/query/acc.cgi?acc=GSE182127). Repository name: Zenodo Permanent identification number: 10.5281/zenodo.5638720 Direct link to the dataset: https://doi.org/10.5281/zenodo.5638720.
Related research article	Buzzi, R. M., Akeret, K., Schwendinger, N., Klohs, J., Vallelian, F., Hugelshofer, M., & Schaer, D. J. (2021). Spatial transcriptome analysis defines heme as a hemopexin-targetable inflammatoxin in the brain. *Free Radical Biology and Medicine*. https://doi.org/10.1016/j.freeradbiomed.2021.11.011

## Value of the Data

• The hemoglobin-derived secondary red blood cell toxin heme is believed to represent a key factor in the multifactorial pathophysiology of secondary brain injury after intracranial hemorrhage [2], [3], [4]. The published dataset provides spatially resolved information on the dose-dependent transcriptional changes in the mouse brain after exposure to heme with or without the heme-scavenger hemopexin.
• Our dataset can be used without modification by basic and translational scientists in the field of hemorrhagic stroke.
• This dataset can be further explored to identify specific molecular pathways or target genes of heme derived toxic effect in the brain, which might direct the development of novel biomarkers or therapeutic agents targeting secondary brain injury after intracranial hemorrhage.
• Furthermore, the herein provided heme-specific transcriptional signature can be reused to identify heme-derived toxic effects in complex datasets from patients after intracranial hemorrhage (e.g. bulk sequencing or single cell data), thereby allowing to further deconvolute the complex secondary pathophysiological processes induced after intracranial hemorrhage.

## 1 Data Description

**Fig. 1:**
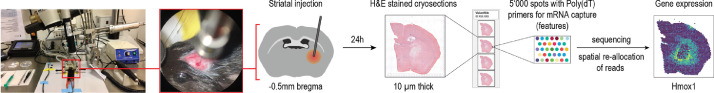
Experimental setup. 24 h after stereotactic injection of 10 µL heme, heme-hemopexin, or saline into the right striatum, mice were euthanized and brain harvested for coronal cryosections, which were placed on a Visium slide to obtain spatial RNA sequencing data. Modified from [1].

**Fig. 2:**
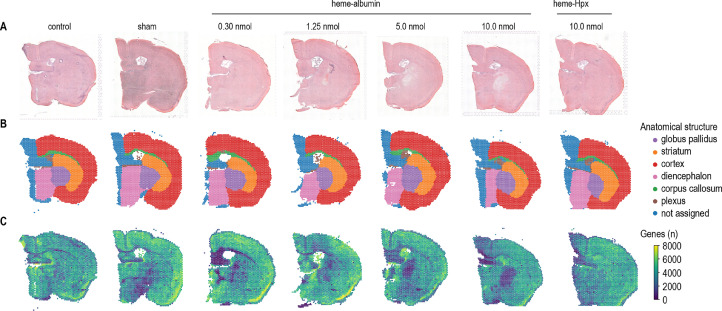
Spatial RNA sequencing dataset structure. (A) H&E images of the seven samples were used to obtain the spatial transcriptome. (B) Histology-based manual segmentation of features into different anatomical structures for stratified downstream analysis. (C) Representation of overall gene count per feature. Modified from [1].

**Fig. 3:**
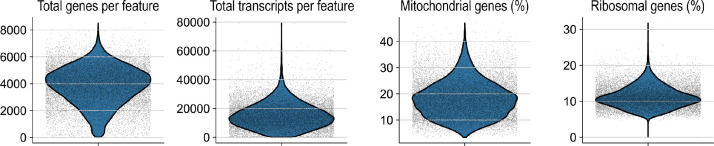
Quality control metrics. Violin plots visualizing the number of total genes, total transcripts per feature, and the percentage of mitochondrial and ribosomal genes across all seven datasets.

We have recently defined heme as a potent hemopexin-targetable inflammatoxin in the brain [1]. Here we provide the spatial RNA sequencing (spRNAseq) datasets characterizing the dose-dependent transcriptional brain response to heme and complexes of heme-hemopexin, respectively. We injected a volume of 10 µL into the right striatum under stereotactic guidance using a motorized frame and a microinjection pump (Fig. 1). A heme dose-response was assessed by exposing mouse brains (striatal injection) to increasing amounts of heme, using 0.30, 1.25, 5.00, and 10 nmol of heme. The highest dose corresponds to approximately 10% of the total heme in a 10 µL-sized hematoma. Additionally, we injected 10 nmol of heme in complex with the endogenous scavenger protein hemopexin (heme:Hpx). The injection of 10 µL saline and an untouched mouse brain served as controls. 24 h after injection, mice were perfused with ice-cold phosphate-buffered saline, and brains were harvested, trimmed, and immediately frozen using liquid nitrogen. Then the samples were sliced into 10 µm thick lesion-centered coronal sections and placed onto the active surface of Visium slides (10X Genomics, Pleasanton, CA, USA). Widefield microscopic images of the sections were obtained after H&E staining (Fig. 2A), followed by mRNA extraction, library construction, and sequencing. The reads were mapped into the two-dimensional space using the unique spatial barcodes that are integrated into the poly(dT) primers used for mRNA capture. In total, we obtained seven individual spRNAseq datasets representing seven different conditions (control, sham, 0.30, 1.25, 5.00, 10 nmol heme, 10 nmol heme:Hpx). The raw data and the Space Ranger output were deposited on the Gene Expression Omnibus and are available under the Gene expression omnibus ascension number GSE182127 (https://www.ncbi.nlm.nih.gov/geo/query/acc.cgi?acc=GSE182127). Based on the H&E image, two independent researchers assigned each feature of the samples to one of six anatomical regions (globus pallidus, striatum, cortex, diencephalon, corpus callosum, and plexus). This anatomical segmentation of the dataset supports region-specific expression analysis (Fig. 2B). Basic quality control analysis revealed comparable counts between samples, except for the central lesion, which showed lower gene counts (Fig. 2C). Across the seven spRNAseq datasets, the median number of detected genes per feature was 3989.5, the median number of transcripts was 14142.5, while the percentage of mitochondrial genes and ribosomal genes per feature was 17.9% and 11.1%, respectively (Fig. 3). To allow for a comparison of spatial gene expression between different conditions and to perform downstream analysis, we provide three additional spRNAseq datasets consisting of merged data from the individual conditions. First, we created a dataset combining the animals injected with 10.0 nmol heme, the vehicle control (sham), and the untouched control (filename: Heme_vs_ctrl_vs_sham.h5ad). Second, we created a dataset with the different heme doses covering the full heme-exposure range (control, sham, 0.30, 1.25, 5.00, 10 nmol heme, filename: Heme_dose.h5ad). Third, we created a dataset containing the spRNAseq data after injection of 10.0 nmol heme and 10.0 nmol heme:Hpx (filename: Heme_vs_hemeHpx.h5ad). All merged datasets, the input data, and the python notebooks to reproduce the basic data analysis and the dataset merging are available on Zenodo (https://doi.org/10.5281/zenodo.5638720).

## 2 Experimental Design, Materials and Methods

### 2.1 Mouse striatal injection model

Wild-type male mice (C57BL/6J, 10-12 weeks old) from Charles River Laboratories (Sulzfeld, Germany) were used for striatal injections. They were housed in individually ventilated cages at the animal facility of the University of Zurich with a 12/12 h dark/light cycle. Prior to the start of the experiment, they were acclimatized to the animal facility for at least two weeks. For the surgery, the mice were anesthetized with isoflurane (Baxter Healthcare Co. Deerfield, IL, USA). For induction, mice were placed in an induction box and exposed to 5% isoflurane evaporated in 100% oxygen, which was subsequently lowered to 1-2% for maintenance targeting a respiratory rate of 60/min as a correlate for a sufficient anesthesia depth. Anesthetized animals received Temgesic i.p. (0.1 mg/kg) as pre-emptive analgesia and were placed in a motorized stereotaxic frame (Stoelting, Dublin, Ireland) and fixed with earbars. During the surgical procedure, the body temperature was monitored with a rectal probe and maintained constant at 37 °C with the use of an electronic thermostat-controlled warming blanket. To protect the eyes for the duration of the surgery Vitamin A cream was applied. After animal placement, the substance to be injected (heme-albumin, heme-hemopexin or saline [0.9 %; B.Braun, Melsungen, Germany]) was filled into the syringe (NANOFIL-100, world precision instruments, Sarasota, FL, US) of the injection pump. Then as the first step in the surgical procedure, with a scalpel a 1 cm long midline skin incision was made. To expose the skull, the skin was mobilized with tweezers by blunt preparation and the soft tissue covering the skull was removed using sterile cellulose swab (MediSet Cellodent 4 × 5 cm steril; IVF Harmann, Neuhausen, Switzerland). After correct horizontal leveling of the skull, the tip of the 33-gauge needle from the syringe attached to the stereotaxic frame was moved to bregma and the atlas referenced to bregma. Then the frame was moved above the entry point for the target. With a scalpel the position was marked, the syringe retracted 3-5 mm and a small burhole was drilled. Then the syringe with the 33-gauge needle was slowly advanced (0.1 mm/s) into the striatum (bregma coordinates: 2 mm ML, 0.5 mm AP, 3.5 mm DV). After reaching the target 1-2 uL of histoacryl (B. Braun Medical AG, Sempach, Switzerland) were applied around the needle in the burrhole to prevent reflux. Then 10 µL of the experimental solution were injected with an infusion rate of 1000 nL/min, resulting in a total injection duration of 10 min. The needle was left in place for 5 min, followed by slow retraction (0.1 mm/s). If relevant bleeding occurred after retraction of the needle caused by an accidental laceration of a vessel during the injection procedure, the animal was excluded from the experiment. Otherwise the burhole was closed with 1-2 µL histoacryl and the skin incision closed with a suture and the animals were placed into a heated wake-up box and monitored until conscious and fully recovered. After recovery, mice were placed back into their home cage. For each condition, two mice were injected and randomly chosen for sectioning with subsequent sequencing and analysis.

### 2.2 Heme-albumin and heme-hemopexin solutions

The heme-albumin solution was prepared as previously described [5]. 61.65 mg hemin (H65195G, Frontier Scientific, Logan, UT, USA) was dissolved in 10 ml of NaOH (100 mM) at 37 °C, followed by the addition of 10 ml 20% human serum albumin (CSL Behring, Bern, Switzerland) and incubation at 37 °C for 60 min. Then, the pH of the solution was adjusted to 7.4 using ortho-phosphoric acid, and the final volume was brought to 25 ml with saline solution. The resulting heme-albumin solution (4 mM) was sterile-filtered (0.22 µm), diluted with saline, and used immediately. Human Hpx was provided by CSL Behring (Bern, Switzerland), and the heme-Hpx complex solution was prepared by adding an equimolar amount of heme. Solutions were always prepared immediately prior to surgery and stored on ice until being drawn into the injection syringe.

### 2.3 Brain harvesting and freezing

24 h after injection, mice were transcardially perfused and the brain harvested. Therefore, mice were deeply anesthetized by intraperitoneal injection of an anesthesia mix containing ketamine (80 mg/kg) + xylazine (16 mg/kg) + acepromazine (3 mg/kg). Depth of the anesthesia was assessed by overvation of the paw withdrawal reflex. Absence of a reaction to a toe pinch was considered a sufficient depth of anesthesia. Then the mice were fixed at all four extremities on a dissecting pan (Carolina Biological Supply Co, Burlington, NC, USA) and the heart exposed by a lateral incision through the integument and abdominal wall just below the xiphoid process followed by two cuts through the rib cage on either side up to the collar bone. The tip of the sternum was lifted away and fixed above the head with a needle. Then a 22-gauge blunt needle (922050-TE; Metcal, Cypress, CA, USA) was inserted into the left ventricle, followed by a small incision into the right atrium. Then the animal was perfused with with 50 ml of pre-cooled (4 °C) phosphate-buffered saline (PBS; Thermo Fisher Scientific, Waltham, MA) using a peristaltic pump (BT50S Microflow Variable-Speed Peristaltic Pump with a YT15 Pump Head; Golanderpump, Norcross, GA) with a rate of 10 mL/min. After completion of transcardial perfusion, the mouse was decapitated with a scissor and the brain carefully extracted. Therefore, a longitudinal cut along the sagittal suture was made with a scalpel to expose the skull. With small surgical scissors two longitudinal cuts were from the foramen magnum to lambda and from the interocular region to bregma, always carefully sliding the scissors along the internal table of the skull to prevent damage to the brain by lifting up the tip. Using tweezers, the skull was then unfolded on both sides exposing the brain. The cranial nerves were carefully cut with scissors and the brain removed. With a brain matrix (Acrylic Mouse Brain Slicer Matrix BSMAA001-1; Zivic Instruments, Pittsburgh, PA) the first 3 mm of the anterior brain, including the olfactory bulb, were trimmed away. Then the trimmed brain piece was immediately flash-frozen in cold Tissue-Tek OCT Compound (Sakura Finetek, Alphen aan den Rijn, Netherlands) using liquid nitrogen-cooled isopentane and stored at −80 °C until sectioning. The time from completion of perfusion until freezing was maintained below 1 min.

### 2.4 Tissue sectioning

Using a Leica CM3050 S Cryostat (Leica, Germany), 10 µm lesion-centered coronal cryosections were prepared and placed within the etched frames of the capture areas on the active surface of the Visium Spatial Slide (10X Genomics, Pleasanton, CA, USA). Prior to sectioning all material used was placed into the cryostat and allowed to equilibrate for at least 15 min. Best results were obtained with object temperature set to −10 °C and chamber temperature set to −15 °C, however, these settings may vary depending on the cryostat and personal preference. As the fiducial frame is barely visible in the cryostat chamber, it was marked on the back side of the Visium Spatial Slide using a permanent marker (Lumocolor permanent pen 313 black; STAEDTLER Mars GmbH & Co. KG, Nuernberg, Germany). As pens do not work at low temperatures, this has to be done prior to introduction of the slide into the cryostat chamber. This additional step not mentioned in the approved protocol efficiently improved placement of the cryosections, which can be easily trained using standard glass slides with marks representing the same layout as the Visium spatial slide. After sequential placement of the different samples onto the capture areas, the slides containing the mounted tissue sections were stored on dry ice in a closed slide container until further processing.

### 2.5 Visium workflow-staining, mRNA extraction, library preparation and sequencing

After placement, the tissue was processed exactly following the manufacturer's instructions (User Guide Visium spatial gene expression, CG000239 Rev; provided as supplemental material). Using the adaptor provided by 10x genomics the slides were heated at 37 °C for 1 min on a T100 thermal cycler (BioRad, Hercules, CA, USA) and then immediately transferred to precooled methanol (−20 °C, ≥99.9%, Sigma Aldrich, St. Louis, MO) for 30 min. After fixation, the sections were stained with H&E using a standard protocol as follows: Sections were incubated with isopropanol (1 min; Sigma Aldrich, St. Louis, MO), then dried at room temperature (10 min), followed by hematoxylin (7 min; Hematoxylin, Mayer's (Lillie's Modification), S330930-2, Agilent), Bluing Buffer (2 min; Dako, CS70230-2, Agilent) and Eosin (1 min, Eosin Y solution, aqueous, 0.5% (w/v) in water, HT110216-500 ML, Millipore Sigma). Between the individual steps, the slides were washed with Milli-Q water. Then, the sections were dried at 37 °C for 5 min. Brightfield images of the H&E stained sections, including the fiducial frames, were obtained using an AxioObserver (Zeiss, Germany) prior to permeabilization. Subsequently, stained sections were strictly processed as outlined in the demonstrated protocol (User Guide CG000239, Rev A). In brief, the tissue was permeabilized for 6 min, as experimentally determined using the Visium Spatial Tissue Optimization Slide & Reagent kit (10X Genomics, Pleasanton, CA, USA). After permeabilization, the released mRNA, bound to oligonucleotides on the capture areas. This was followed by reverse transcription, second-strand synthesis, denaturation and cDNA amplification. All steps were exactly carried out as specified in the demonstrated protocol using a T100 thermal cycler with the spatial slide adaptor. Step 4.1 in the protocol to define the optimal number of cycles used for cDNA amplification was performed on a C1000 Touch Thermal Cycler coupled to a CFX96 Touch Real-Time PCR Detection System (Biorad, Hercules, CA, USA). Accordingly, the number of cycles for cDNA amplification used in Step 4.2 was set to 15. After amplification, cDNA clean-up using SPRIselect (Beckman Coulter, Brea, CA, USA) and library preparation was performed strictly adhering to the protocol. Then, cDNA libraries were sequenced on an Illumina NovaSeq 6000 (Illumina, San Diego, CA, USA) by the Functional Genomic Center Zurich (FGCZ, Zurich, Switzerland) with 150 cycles as follows: 28bp (Read 1), 10bp (i7 Index), 10bp (i5 Index), 120bp (Read 2).

### 2.6 Alignment, quantification, and quality control of Visium data

The alignments of reads, filtering, barcode counting, and UMI counting were performed using Space Ranger 1.1.0 (10X Genomics, Pleasanton, CA, USA) and the FASTQ files and tiff files of the H&E histology as input. The reads were aligned to the Genome Reference Consortium Mouse Build 38 patch release 6 (GRCm38.p6). Space Ranger additionally reallocated the barcodes in each read with the respective 55 µm feature (i.e., single spots on the slide with a distinct spatial position defined by a unique barcode) relative to the fiducial frame. This associates each read count with the spatial location and the histological image.

### 2.7 Anatomical segmentation

Using the Loupe Browser (version 5.0, 10X Genomics, Pleasanton, CA, USA), the features of each sample were manually segmented into six anatomical regions (globus pallidus, striatum, cortex, diencephalon, corpus callosum, and plexus) based on the H&E image using the Allen Brain Atlas [6] as a reference. Then the anatomical feature annotation was exported as a csv file using the export option in the Loupe Browser.

### 2.8 Downstream analysis of 10x genomics Visium data

The processed spRNAseq data were analyzed with the Scanpy python package (version 1.4.4) [7]. A standard processing pipeline according to the tutorial on spatial transcriptomics data analysis for quality control, visualization, and downstream analysis. The individual spRNAseq datasets were merged using the Scanorama package (version 1.7) [8] to compare the different conditions, thus creating three new datasets. After merging, the data were normalized using a scaling factor of 1000 and log-transformed. Variable genes with “Seurat flavor“ were detected, and a principal component analysis was performed. Finally, a neighborhood graph was built, and Uniform Manifold Approximation and Projection (UMAP) was calculated to visualize the data. Clusters were defined using the Leiden algorithm [9]. The entire code used to reproduce the basic processing and analysis based on the raw data is available as a jupyter notebook on Zenodo (https://doi.org/10.5281/zenodo.5638720).

## Ethics Statements

All animal experiments performed complied with the ARRIVE guidelines and were reviewed and approved by the Veterinary Office of the Canton of Zurich (ZH89/2019). To reduce variability, only male animals were used in this study.

## CRediT authorship contribution statement

**Kevin Akeret:** Conceptualization, Methodology, Investigation, Validation. **Michael Hugelshofer:** Conceptualization, Supervision. **Dominik J. Schaer:** Conceptualization, Resources, Funding acquisition, Supervision. **Raphael M. Buzzi:** Conceptualization, Methodology, Investigation, Software, Funding acquisition, Validation.

## Declaration of Competing Interest

RMB, KA, MH, and DJS are inventors on a provisional patent application for the use of Hpx in aneurysmal subarachnoid hemorrhage.

## Appendix Supplementary Materials

User Guide - Visium Spatial Gene Expression Reagent Kits
